# Cryptosporidiosis in renal transplant recipients; concern on effective diagnosis

**Published:** 2015-07-03

**Authors:** Beuy Joob, Viroj Wiwanitkit

**Affiliations:** ^1^Sanitation1 Medical Academic Center, Bangkok Thailand; ^2^Hainan Medical University, China

**Keywords:** Cryptosporidiosis, Renal, Transplantation

**Implication for health policy/practice/research/medical education:**

Various fungal infections pose significant problems for the renal transplant recipients in terms of morbidity and mortality. Of several fungal infections, cryptosporidiosis is an important infection to be managed. Its early diagnosis and management are essential in renal transplant patients. A high index of suspicion and use of newer diagnostic tests are warranted.

Opportunistic infections among the renal transplant recipients are very interesting and pose diagnostic and therapeutic challenges. Several uncommon infections can be seen. Opportunistic fungal infections are a common occurrence in this group of patients. Various fungal infections become medical problems for the renal transplant recipients. Of several fungal infections, cryptosporidiosis is an important infection to be managed. Here, we would like to discuss on the recent report on “cryptosporidiosis in renal transplant recipients” from a single center in Pakistan published in Journal of Nephropathology (1). Raja et al noted that “it is recommended that in all transplant recipients presenting with acute diarrhea, modified ZN staining should be done to rule out cryptosporidiosis (1).” In fact, cryptosporidiosis is an important parasitic infestation that can be seen in immunocompromised hosts including the transplant recipients. In renal transplant patients, long-term renal failure before transplantation can result in chronic uremia which directly suppresses the immune system and the use of immunosuppressive drugs after transplantation can result in more decreased immunity (2). The parasitic infestations, especially cryptosporidiosis, become more prevalent (2). In clinical practice, cryptosporidiosis is an important cause of diarrhea in renal transplant patients (3). Since this infection can be effectively managed if diagnosed early, a high index of suspicion by the practitioner for its diagnosis is important. In diagnosed cases, several antimicrobials including azithromycin, spiramycin, nitazoxanide, or paromomycin are proved to be effective for treatment (4,5). Krause et al also noted that “delay in initiation of treatment can result in serious complications including acute renal failure (6)”.

This infection should be ruled out in any transplant case with gastrointestinal problem. The extremely high (53%) prevalence in the study under discussion (1) can draw attention of practitioner to this parasitic infestation among the renal transplant recipients. However, it should be noted that the diagnostic technique in this study has low sensitivity and specificity (7,8). The use of microscopic examination with special staining has the sensitivity of about 80.9% compared to polymerase chain reaction (PCR) technique (9). Hawash et al noted that the microscopic diagnostic technique was the worst compared to immunological test and PCR (8). In general practice, a more acceptable technique such as sandwich antigen detection enzyme linked immunosorbent assay (ELISA) should be used for more accurate diagnosis (8). Compared to microscopic technique, the reported sensitivity (100%) and specificity (97%) of the immunological test, are more preferable (10). With use of standard sandwich antigen detection ELISA, we might expect higher infestation rate and highlight the importance of the problem for the nephrologist/transplant physician.

## Author’s contribution

BJ conducted literature review and wrote the article. BJ and VW planned and conducted literature review, and finalized it. All authors read and signed the manuscript.

## Conflicts of interest

The authors declared no competing interests.

## Ethical considerations

Ethical issues (including plagiarism, data fabrication, double publication) have been completely observed by the authors.

## Funding/Support

None.
